# Non-pharmacologic techniques for interval intrauterine device placement: a systematic review

**DOI:** 10.1136/bmjsrh-2025-202840

**Published:** 2025-11-03

**Authors:** Tesfaye Tufa, Emily Snyder, Sophia Garbarino, Mekdes Wolderufael, Petrus Schonken Steyn

**Affiliations:** 1 Department of Obstetrics and Gynecology, University of Toronto, Toronto, Ontario, Canada; 2 Noorda College of Osteopathic Medicine, Provo, Utah, USA; 3 Department of Behavioral, Social, and Health Education Sciences, Emory University Rollins School of Public Health, Atlanta, Georgia, USA; 4 Department of Obstetrics and Gynecology, St Paul’s Hospital Millennium Medical College, Addis Ababa, Ethiopia; 5 UNDP/UNFPA/UNICEF/WHO/World Bank Special Programme of Research, Development of Sexual and Research Training in Human Reproduction (HRP Research), Department of Sexual and Reproductive Health and Research, World Health Organization, Geneva, Switzerland

**Keywords:** Contraceptive Agents, Female, Contraceptive Devices, Female, Contraceptive Effectiveness

## Abstract

**Objective:**

To systematically review the evidence on the effectiveness of non-pharmacologic techniques for interval intrauterine device (IUD) placement.

**Methods:**

We searched various databases from database inception to 15 December 2023 for randomised controlled trials (RCTs) examining non-pharmacologic techniques for IUD placement compared with placebo or pharmacologic techniques. Outcomes of interest were pain experienced with IUD placement, provider ease of placement, need for adjunctive placement measures, placement success, patient satisfaction, side effects and adverse events. We extracted data from included articles, assessed risk of bias, and determined certainty of evidence for all outcomes.

**Results:**

Eleven RCTs met the inclusion criteria, examining 10 different non-pharmacologic techniques. The risk of bias was high in 10 trials and low in 1 trial. Of 11 RCTs that examined patient pain, two found reduced pain with the Valsalva breathing technique (where women inhaled deeply and held their breath during IUD placement), without tenaculum placement compared with routine care (i.e., use of a tenaculum) (OR 0.04; 95% CI 0.01 to 0.15) or acupuncture compared with routine care (mean difference −1.88; 95% CI −2.72 to –1.04). None of the four trials that assessed ease of IUD placement found differences between study groups. Across nine RCTs, most had high rates of placement success. One study each examined need for cervical dilation and patient satisfaction; the findings were similar between study groups. Overall, few side effects and no adverse events were reported across the studies.

**Conclusions:**

Non-pharmacologic techniques like the Valsalva manoeuvre and acupuncture may reduce patient pain with IUD placement (certainty of evidence ranging from moderate to low). However, evidence is limited, and more high-quality trials with larger sample sizes are needed.

**Prospero registration number:**

CRD42024507788.

**WHAT IS ALREADY KNOWN ON THIS TOPIC**

- Determining effective and practical approaches to ease intrauterine device (IUD) placement and alleviate patient pain may reduce barriers to IUD access, increasing patient access to comprehensive contraceptive options.

**WHAT THIS STUDY ADDS**

- Little is known about non-pharmacologic techniques for IUD placement.
- This systematic review summarises the available evidence examining the effectiveness of non-pharmacologic techniques used to ease IUD placement and reduce patient pain.

**HOW THIS STUDY MIGHT AFFECT RESEARCH, PRACTICE OR POLICY**

- The limited evidence available examining non-pharmacologic techniques for IUD placement underscores the need for more well-designed, large-scale, randomised controlled trials to strengthen the evidence base.
- Given that many of the non-pharmacologic techniques examined are readily available in most settings and need few resources, and few side effects and no adverse events were reported, clinicians might consider using a combination of pharmacologic and non-pharmacologic strategies tailored to the individual patient’s preferences and needs.

## Background

****Intrauterine devices (IUDs) are highly effective reversible methods of contraception used to prevent pregnancy and optimise interpregnancy spacing.^1^ Providing long-term contraceptive protection with minimal effort for ongoing use after placement, IUDs are the most commonly used reversible form of contraception globally, with approximately 160 million users worldwide.^2^ However, misconceptions and barriers surrounding IUD use persist, preventing some women from accessing them.^3^ As a result, IUD use is limited in some parts of the world, particularly in developing countries.^4^,^6^

One barrier to IUD use is provider concern about potential technical difficulties with IUD placement, particularly for certain groups of women, including adolescents, nulliparous women and women with cervical stenosis due to cervical pathology or previous cervical procedures.^1 7^ Another barrier to IUD use is patient fear of potential pain with IUD placement, leading some patients to choose another contraceptive method.^8^,^10^

Determining effective and practical approaches to ease IUD placement and alleviate patient pain may reduce barriers to IUD access, increasing patient access to comprehensive contraceptive options and promoting reproductive autonomy.^11^ Several systematic reviews have examined the effect of different pharmacologic methods to improve the IUD placement experience without identifying conclusive evidence supporting the effectiveness of specific medications.^12^,^17^ Less is known about the use of non-pharmacologic techniques to improve the IUD placement experience, with no systematic reviews summarising the evidence. The objective of this systematic review was to evaluate the evidence on the effectiveness of non-pharmacologic techniques for interval IUD placement. The review was conducted as part of a process to update the WHO Selected Practice Recommendations,^18^ which provides evidence-based guidance for healthcare providers on the provision of contraceptive methods and the management of side effects and other issues related to contraceptive use.

## Methods

We conducted this review according to a protocol registered in the international Prospective Register of Systematic Reviews (PROSPERO; registration number CRD42024507788)^19^ and reported details according to the Preferred Reporting Items for Systematic Reviews and Meta-Analyses (PRISMA) guidelines.^20^

### Eligibility criteria

** **We included randomised controlled trials (RCTs) that examined non-pharmacologic techniques compared with placebo or with pharmacologic techniques and excluded non-randomised controlled trials, observational studies, case reports, editorials and opinion papers. To focus the review more narrowly, we excluded trials that examined instruments or devices used to facilitate IUD placement (eg, ultrasound guidance, forceps). We included trials that examined placement of currently available levonorgestrel (LNG)-releasing IUDs, or any Copper-T IUD, for women of any age, of any parity and for any indication. However, we only included studies that examined interval IUD placement, defined as placement occurring at least 6 weeks’ postpartum or at least 4 weeks’ post-abortion.

We included trials that examined our primary outcomes: (1) pain experienced with IUD placement at specific time points (ie, during tenaculum placement, during IUD placement, and highest level of pain after placement and before clinic discharge); (2) provider ease of placement; (3) need for adjunctive placement measures (ie, cervical dilation, ultrasound guidance, local anaesthesia, analgesia); and (4) placement success. We also included trials that examined our secondary outcomes: (5) patient satisfaction with the procedure assessed before clinic discharge; (6) side effects occurring before clinic discharge (eg, nausea, vomiting, preplacement abdominal pain or cramping, diarrhoea); and (7) adverse events occurring before clinic discharge (eg, uterine perforation, vasovagal reaction). RCTs are the most appropriate design to study the interventions and outcomes of interest included in this review.

### Literature search and study selection

We systematically searched MEDLINE, EMBASE, Cochrane, WHO Global Index Medicus, Scopus, ClinicalTrials.gov and WHO International Registry platforms using a predetermined comprehensive search strategy (see online supplemental Appendix) from database inception to 15 December 2023. We limited search results to articles that included human subjects only. We optimised our search by also reviewing references in systematic reviews. We considered articles in all languages for inclusion. Two investigators reviewed titles and abstracts to identify studies examining non-pharmacologic techniques to ease IUD placement; any conflicts identified during study selection were resolved by discussion.

### Data extraction, risk of bias assessment, data synthesis and certainty of evidence

Two investigators extracted data from each included study and assessed the risk of bias for each trial following the guidelines in the Cochrane Handbook of Systematic Reviews of Interventions.^21^ Any disagreements were resolved through discussion. Using the Cochrane guideline risk of bias assessment tool, we evaluated the risk of bias for included RCTs across six domains: selection bias (random sequence generation and allocation concealment), blinding of participants and personnel, blinding of outcomes assessment, attrition bias, reporting bias and other sources of bias.^21^ We conducted a narrative synthesis of the included studies and used RevMan 2014 to calculate effect estimates. Due to the diversity of the included studies, it was not possible to conduct a pooled analysis. We used the GRADE (Grading of Recommendations, Assessment, Development and Evaluations) approach to assess the certainty of evidence for our primary outcomes.^22^ For each outcome, we evaluated risk of bias, directness, precision and consistency to be not serious, serious or very serious. Based on these assessments, the certainty of evidence was rated as high, moderate, low or very low.

## Results

**Figure 1:**
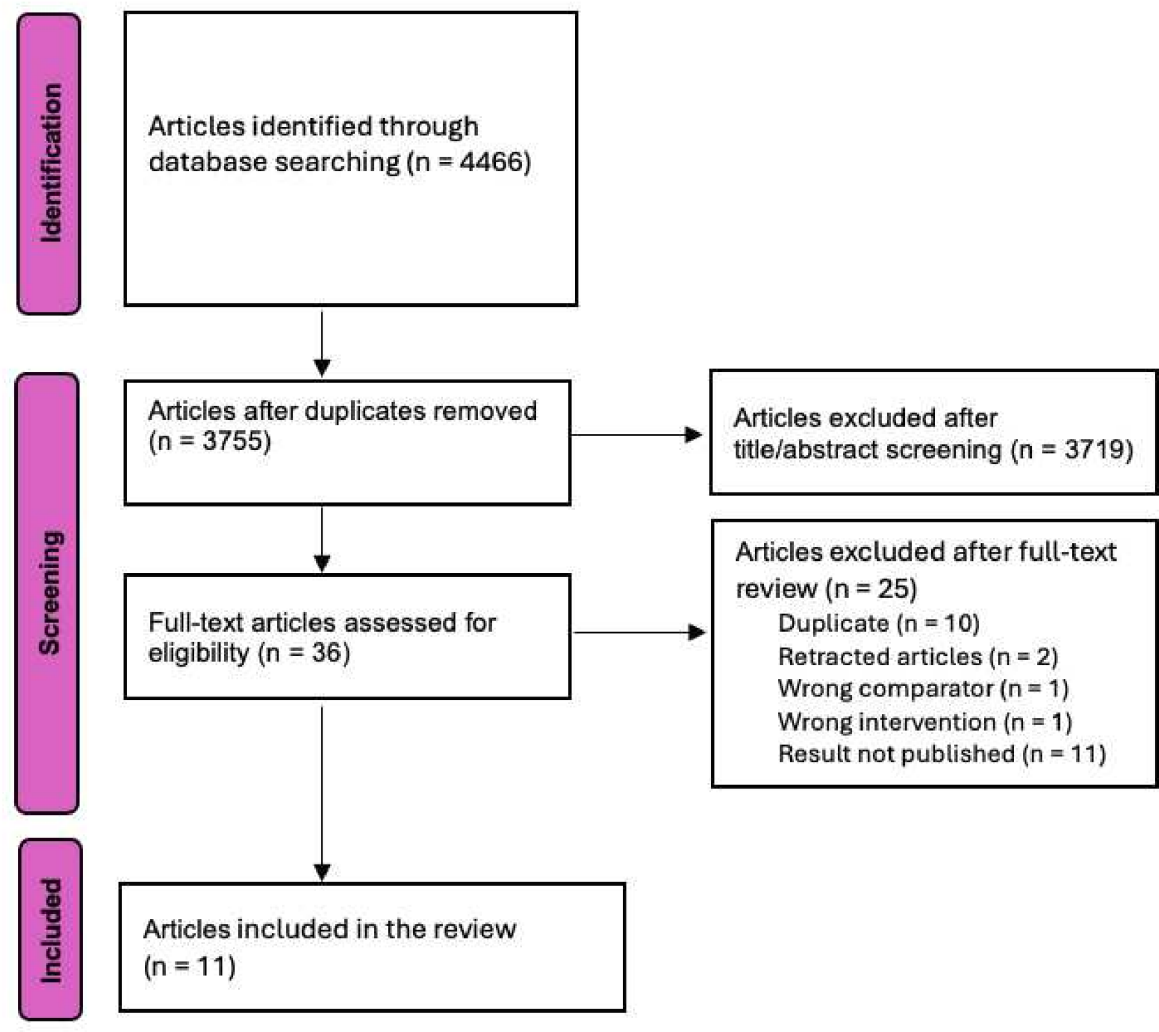
PRISMA (Preferred Reporting Items for Systematic Reviews and Meta-Analyses) flow diagram for the search process for evidence on non-pharmacologic techniques to ease interval intrauterine device placement.

The search yielded 4466 articles, of which 11 RCTs met the inclusion criteria (figure 1). The 11 included trials examined various non-pharmacologic techniques used to facilitate IUD placement in a total of 1286 women (table 1). Two studies examined inhaled lavender during IUD placement.^23 24^ Two studies examined IUD placement at different times of the menstrual cycle; one compared placement during menstruation with placement outside of menstruation^25^ and the other compared placement at different menstrual cycle segments calculated by dividing the menstrual cycle day of placement by the total number of days in the menstrual cycle.^26^ One study each examined acupuncture before IUD placement;^27^ verbal analgesia, which consisted of provider reinforcement and calming before each step during IUD placement;^28^ use of a virtual reality headset where women were offered to choose one of four virtual universes (forest, meadow, ocean floor or outer space);^29^ cold compression on the lower abdomen for 5 min before and during IUD placement;^30^ the Valsalva manoeuvre, a breathing technique where women inhaled a deep breath and held it during IUD placement;^31^ the cough method whereby participants were asked to give one strong cough during which the tenaculum was placed on the cervix;^32^ and delayed bladder emptying whereby participants drank 1 litre of fluid in the hour before the appointment and emptied their bladder only after IUD placement.^33^

**Table 1:** Characteristics of included randomised controlled trials: systematic review of non-pharmacologic techniques to ease interval intrauterine device placement

Reference, year, location	Participants	Non-pharmacologic intervention	Description of intervention	Control group	Outcomes reported	Results
Aliei *et al*., 2013^23^ RCT Multicentre, Iran	N=135 Age of participants not specified	Lavender oil (group A)	Participants asked to inhale lavender oil for a few minutes before IUD placement	Sesame oil (placebo) (group B) or routine care (no treatment) (group C)	**Primary outcomes** Pain experienced with IUD placement assessed using 10-point scale (0=no pain; 10=the worst pain) **Secondary outcomes** Side effects occurring before clinic discharge (%)	**Pain experienced with IUD placement** (mean (SD), n): A: 2.60 (2.71), n=45 B: 4.57 (2.71), n=45 C: 3.82 (2.08), n=45 Comparison of lavender (A) and sesame oil (B) (p<0.001); MD=−1.97; 95% CI −3.09 to –0.85 Comparison of lavender (A) and control group (C) (p<0.004); MD=−1.22; 95% CI −2.22 to –0.22 **Side effects occurring before clinic discharge**: No side effects observed
Benazzouz *et al*., 2024^29^ RCT Two sites Rennes, France	N=100 Aged ≥18 years	VR headset (group A)	VR headset offered when clients were positioned on examination table and ready for IUD placement. Women could choose one of four virtual universes (forest, meadow, ocean floor or outer space)	Routine care with no VR headset (group B)	**Primary outcomes** Pain experienced with IUD placement assessed using 10-point scale (0=no pain; 10=the worst pain) Placement success **Secondary outcomes** Patient satisfaction with the procedure assessed using 10 cm scale (0=not satisfied; 10=completely satisfied)	**Pain experienced with IUD placement** (p=0.54) (mean (SD), n): A: 5.1 (2.5), n=47 B: 5.4 (2.7), n=48 MD=−0.30; 95% CI −1.35 to 0.75 **Pain immediately after procedure** (p=0.98) (mean (SD), n): A: 2.4 (2.2), n=47 B: 2.4 (2.3), n=48 MD=0.00; 95% CI −0.90 to 0.90 **Placement success** (n/N, %): A: 47/50, 94% B: 48/50, 96% **OR=0.65; 95% CI 0.1 to 4.09** **Patient satisfaction with the procedure** (p=0.87) No difference between groups (mean 9.6 in both groups)
Cameron *et al*., 2013^33^ RCT Single centre, UK	N=200 Age of participants not specified	Delayed bladder emptying (group A) Age of participants not specified	Women drank 1 L of fluid in the hour before the appointment and emptied their bladder only after IUD placement	Immediate bladder emptying before IUD placement (group B)	**Primary outcomes** Pain experienced with IUD placement assessed using 10-point scale (0=no pain; 10=agony) Provider ease of placement assessed using five-point scale from “very easy” to “very difficult” and dichotomised (% very or quite easy) Placement success (%)	**Pain experienced with IUD placement** (p=0.1) (mean (SD), n): A: 3.8 (2.3), n=97 B: 4.4 (2.3), n=99 MD=−0.60; 95% CI −1.24 to 0.04 **Provider ease of placement** (p=0.68) (n/N, %): Reported very or quite easy placement A: 82/97, 83% B: 83/99, 82% OR=1.05; 95% CI 0.5 to 2.31 **Placement success** (n/N, %): A: 97/100, 97% (3 failures due to cervical stenosis) B: 99/100, 99% (1 failure; woman felt unwell after uterine sound placement) OR=0.33 95% CI 0.03 to 3.19
Cimsir & Yildiz, 2021^31^ RCT Single centre, Turkey	N=107 Aged ≥18 years	Valsalva manoeuvre (VM) without tenaculum placement (group A)	Breathing technique where women inhaled a deep breath and held it during IUD placement without tenaculum placement	Tenaculum application (considered as routine care) (group B)	**Primary outcomes** Pain experienced with IUD placement assessed using 10 cm VAS (0=no pain; 10=severe pain), then categorised as no pain, mild/moderate pain or severe pain; and any pain Placement success (%)	**Pain experienced with IUD placement** (p=0.01) (n/N (%) for A vs n/N (%) for B): No pain: 30/52 (57.7%) vs 3/55 (5.5%) Mild or moderate pain: 22/52 (42.3%) vs 20/55 (36.4%) Severe pain: 0/52 (0%) vs 32/55 (58.2%) Any pain: 22/52 (42.3 %) vs 52/55 (94.5%) OR=0.04; 95% CI 0.01 to 0.15 **Placement success** (p=0.59) (n/N, %) A: 45/52, 86.5% B: 49/55, 89.1% OR=0.79, 95% CI 0.25 to 2.52
Daykan *et al*., 2021^28^ RCT Multicentre, Israeli	N=54 Aged ≥18 years	Verbal analgesia (group A)	Provider reinforcement and calming before each step during IUD placement	Oral tramadol (group B)	**Primary outcomes** Pain experienced with IUD placement assessed using 10 cm VAS (0=no pain; 10=worst pain ever) Provider ease of placement (tool not described) Placement success (%) **Secondary outcomes** Adverse events occurring before clinic discharge (%) Side effects (not specified) occurring before clinic discharge (%)	**Pain experienced with IUD placement** (p=0.526) (mean (SD), n): A: 4.8 (2.4), n=32 B: 4.5 (1.6), n=22 MD = 0.3, 95% CI −0.77 to 1.37 **Provider ease of placement** (p=0.771) (mean (SD), n): A: 6.3 (2.5), n=32 B: 4.5 (1.4), n=22 MD=1.80; 95% CI 0.75 to 2.85 **Placement success**: 100% for both study groups **Adverse events occurring before clinic discharge** (n/N for A vs n/N for B): Syncope: 0/32 vs 1/22 **Side effects occurring before clinic discharge:** Vaginal bleeding requiring packing: 0/32 vs 1/22
Erdoğan & Yardımcı, 2023^27^ RCT Single centre, Turkey	N=72 Aged ≥18 years	Acupuncture (group A)	One session of bilateral LI4 acupuncture before IUD placement	Routine care without acupuncture (group B)	**Primary outcomes** Pain experienced with IUD placement assessed using 10 cm VAS (0=no pain; 10=worst pain ever) Need for adjunctive placement measures (%) Placement success (%) **Secondary outcomes** Side effects occurring before clinic discharge (%)	**Pain experienced 3 min after IUD placement** (p<0.001) (mean (SD), n): A: 1.93 (1.68), n=36 B: 3.81 (1.95), n=36 MD=−1.88; 95% CI −2.72 to –1.04 **Need for cervical dilation** (n/N): A: 2/36 B: 1/36 **Placement success** (n/N, %): A: 36/36, 100% B: 35/36, 97% **Side effects occurring before clinic discharge** (n/N for A vs n/N for B): Prolonged cervical tamponade for bleeding from tenaculum site: 1/36 vs 0/36 Nausea/vomiting: 2/36 vs 2/36
Hocaoğlu *et al*., 2021^26^ RCT Single centre, Turkey	N=192 Aged ≥18 years	IUD placement at different menstrual cycle segments Group A: IUD placed at 0.5 to 0.69 time segment	Placement at different time segments of the menstrual cycle (calculated by dividing menstrual cycle day of IUD placement by total number of cycle days)	Group B: IUD placed at 0.7 to 0.89 time segment Group C: IUD placed at 0.9 to 1.0 time segment	**Primary outcomes** Pain experienced with IUD placement assessed using Wong–Baker FACES Pain Rating Scale Provider ease of placement (% easy, mildly difficult, moderately difficult, very difficult) Placement success (%) **Secondary outcomes** Adverse events occurring before clinic discharge (%)	**Pain experienced during tenaculum placement** (p=0.445) (median (range), n): A: 4 (2-6), n=53 B: 2 (0–6), n=67 C: 4 (2-8), n=72 **Pain experienced during IUD placement** (p=0.505) (median (range), n): A: 4 (0–8), n=53 B: 4 (2-10), n=67 C: 4 (0–6), n=72 **Pain experienced 5 min after IUD placement** (p=0.248) (median (range), n): A: 0 (0–2), n=53 B: 0 (0–2), n=67 C: 0 (0–8), n=72 **Provider ease of placement** (p=0.149): (% for A vs % for B vs % for C): Easy: 50% vs 51.2% vs 42.5% Mildly difficult: 36.8% vs 29.3% vs 40% Moderate difficulty: 10.5% vs 17.1% vs 12.5% Difficult: 2.6% vs 2.4% vs 5% **Placement success:** 100% for all three study groups **Adverse events occurring before clinic discharge**: No adverse events observed
Hylton *et al*., 2020^30^ RCT Two centres, USA	N=142 Aged ≥18 years	Cold compression (group A)	Cold compress placed on abdomen for 5 min before and during IUD placement	Routine care without cold compress (group B)	**Primary outcomes** Pain experienced with IUD placement assessed using 10-point VAS (0=no pain; 10=worst pain imaginable) Placement success (%)	**Pain experienced with IUD placement (p=0.805**) (mean (SD), n): A: 4.3 (2.6), n=72 B: 4.6 (2.5), n=66 MD=−0.30, 95% CI −1.15 to 0.55 **Placement success:** 100% for both study groups
Lambert *et al*., 2020^32^ RCT Single centre, USA	N=66 Aged ≥18 years	Slow method (group A)	Closure of tenaculum slowly over 5 seconds	Cough method (closure of tenaculum at the time of cough) (group B)	**Primary outcomes** Pain experienced with IUD placement assessed using 100 mm VAS (0=no pain; 10=worst pain imaginable) Placement success (%)	**Pain experienced with tenaculum placement** (p=0.16) (median (IQR), n) A: 44 (21, 63), n=33 B: 32 (IQR=19, 54), n=33 **Pain experienced after IUD placement** (p=0.12) (median, IQR, n): A: 62 (IQR=48, 84), n=33 B: 54 (32, 71), n=33 **Placement success:** 100% for both study groups
Shahnazi *et al*., 2012^24^ RCT Single centre, Iran	N=106 Aged ≥15 years	Lavender oil (group A)	Participants asked to inhale lavender oil for few minutes before IUD placement	Placebo (group B)	**Primary outcomes** Pain experienced with IUD placement assessed using 10 cm VAS (0=no pain; 10=very severe pain) Placement success (%) **Secondary outcomes** Side effects occurring before clinic discharge (%)	**Pain experienced with IUD placement** (p=0.51) (median (IQR), n) A: 1 (0–3), n=33 B: 1 (0–3), n=33 **Placement success:** 100% for both study groups **Side effects occurring before clinic discharge**: No side effects observed
van der Heijden *et al*., 2017^25^ RCT Single centre, Netherlands	N=112 Aged ≥18 years	IUD placement at different times of the menstrual cycle	Placement outside of menstruation with no presence of vaginal bleeding (group A)	Placement during days 1–7 of menstruation (group B)	**Primary outcomes** Pain experienced with IUD placement assessed using 100 mm VAS (endpoints not described) Provider ease of placement (% easy)	**Pain experienced with IUD placement for nulliparous women** (p=0.14) (mean (SD), n): A: 66 (20.2), n=28 B: 74 (18.8), n=30 MD=−8.00, 95% CI −18.06 to 2.06 **Pain experienced with IUD placement for multiparous women** (p=0.08) (mean (SD), n): A: 43 (26.9), n=29 B: 30 (24.9), n=25 MD=13.00, 95% CI −0.82 to 26.82 **Provider ease of placement** for nulliparous women (p=0.58) (n/N, %): A: 25/28 (89.3%) B: 28/30 (93.3%) OR=0.60, 95% CI 0.09 to 3.86 **Provider ease of placement** for multiparous women (p=0.10) (n/N, %): A: 26/29 (89.6%) B: 25/25 (100%)

IUD, intrauterine device; MD, mean difference; OR, odds ratio; RCT, randomised controlled trial; SD, standard deviation; VAS, visual analogue scale; VM, Valsalva manoeuvre; VR, virtual reality.

Studies used a variety of different comparison groups. One study compared the non-pharmacologic technique with a medication (tramadol) used before IUD placement^28^ and another compared the non-pharmacologic technique with use of a tenaculum (considered as routine care) for IUD placement.^31^ One study included two control groups (placebo and no treatment)^23^ and another examined three variations of the non-pharmacologic technique (ie, placement at different menstrual cycle segments).^26^ The other seven studies compared the non-pharmacologic technique with placebo or no treatment.

In eight studies,^25^,^32^ women aged 18 years or older were included. One study included patients aged 15 years or older.^24^ The inclusion criteria of two studies^23 33^ did not specify any age restrictions.

Three studies were conducted in Turkey.^26 27 31^ Two studies were conducted in Iran,^23 24^ and two other studies were conducted in the USA.^30 32^ One study each was conducted in: France,^29^ the UK,^33^ Israel^28^ and the Netherlands.^25^

### Risk of bias of individual studies

**Figure 2:**
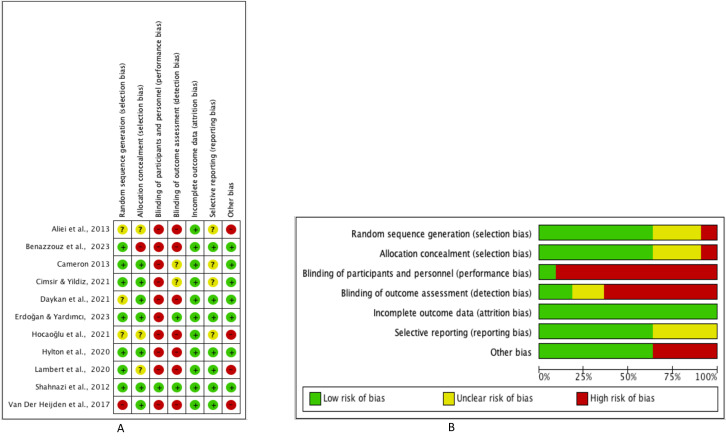
(A) Risk of bias, by domain, for included studies on non-pharmacologic techniques to ease interval intrauterine device placement. Key: low risk of bias (+), high risk of bias (–), unclear risk of bias (?) (B) Risk of bias, by domain, across included studies.

We determined that the risk of bias for one RCT was low,^24^ and the risk of bias for the remaining 10 RCTs was high, mainly due to the lack of blinding of participants and personnel (performance bias) and lack of blinding of outcome assessment (detection bias) (figure 2).

### Effect of intervention

#### Pain experienced with IUD placement

All 11 included studies examined pain experienced with IUD placement. One study measured pain using the Wong–Baker FACES Pain Rating Scale,^26^ and the other 10 studies measured pain using a visual analogue scale (VAS).

One study found that any pain experienced during IUD placement was significantly lower in participants who used the Valsalva breathing technique without tenaculum placement compared with participants in the tenaculum group (odds ratio (OR) 0.04; 95% confidence interval (95% CI) 0.01 to 0.15).^31^ Another study found that pain experienced at 3 min after IUD placement was significantly lower among participants receiving acupuncture before IUD placement compared with those receiving routine care without acupuncture (mean difference (MD) −1.88; 95% CI −2.72 to −1.04).^27^ For lavender oil, whereas one study^23^ found a significant reduction in pain during IUD placement in women who inhaled lavender oil compared with sesame oil (MD −1.97; 95% CI −3.09 to −0.85) and compared with no treatment (MD −1.22; 95% CI −2.22 to −0.22), another similar study^24^ did not demonstrate significant differences between participants inhaling lavender oil and placebo (median 1.0 vs 1.0). In the remaining seven studies that examined pain with IUD placement, no significant differences were found between participants receiving the non-pharmacologic technique compared with placebo or no treatment.^2526 28^,^30 32 33^

#### Provider ease of placement

Four studies examined provider ease of placement,^25 26 28 33^ measured using a Likert scale or as the percentage of providers reporting the IUD placement as easy versus difficult. One study^33^ found no significant differences in provider ease of placement between participants in the delayed compared with early bladder emptying groups (OR 1.05; 95% CI 0.5 to 2.31). Another study^28^ also found no significant differences in provider ease of placement between participants receiving verbal analgesia compared with tramadol (MD 1.80; 95% CI 0.75 to 2.85). In the remaining two studies, both of which examined IUD placement at different times of the menstrual cycle, no significant differences in provider ease of placement between study groups were found^25 26^

#### Need for adjunctive placement measures

Only one study examined the need for adjunctive placement measures, which compared participants receiving acupuncture before IUD placement with those receiving routine care without acupuncture.^27^ In this study, three participants required cervical dilation (two in the acupuncture group (5.6%) and one in the no treatment group (2.8%)).

#### Placement success

Nine studies examined placement success. In five studies, the success rate was 100% across study groups.^24 26 28 30 32^ Four studies reported a small number of placement failures; three of these studies had success rates ≥94% across study groups,^27 29 33^ and one study that examined use of the Valsalva manoeuvre without tenaculum placement versus tenaculum application found success rates of 86.5% in the intervention group compared with 89.1% in the control group (p=0.59).^31^

#### Patient satisfaction

One study examined patient satisfaction with the procedure.^29^ This study^29^ compared satisfaction with IUD placement using a 10 cm numerical scale (0=not satisfied; 10=completely satisfied) between participants using a virtual reality headset compared with those who received routine care with no virtual reality headset; no significant differences were found (mean 9.6 in both groups, p=0.87).

#### Side effects and adverse events

 Five studies reported on side effects or adverse events occurring before clinic discharge, with few side effects or adverse events reported.^2324 26^,^28^ One study that compared acupuncture before IUD placement with routine care without acupuncture^27^ reported nausea and/or vomiting in 5.5% in both the acupuncture and control groups. Additionally, 2.8% of participants in the acupuncture group had prolonged cervical tamponade use due to prolonged bleeding from the tenaculum site; no participants in the control group experienced prolonged bleeding.

 One study reported on side effects and adverse events.^28^ In this study, no patients in the verbal analgesia group developed vaginal bleeding requiring packing, compared with one patient in the tramadol group (4.5%). Related to adverse events, 0% of participants receiving verbal analgesia experienced syncope compared with 4.5% of participants receiving oral tramadol. Another study that examined IUD placement at different menstrual cycle segments examined adverse events but observed none.^26^

 Two studies examined side effects with use of inhaled lavender and found no side effects across study groups.^23 24^

### Certainty of evidence

 For the eight RCTs assessing lavender oil, virtual reality, the Valsalva manoeuvre without tenaculum placement, verbal analgesia, cold compression, slow method of tenaculum closure or IUD placement during menstrual bleeding,^23^,^2528^ the certainty of evidence for all outcomes examined was rated as low, due to serious concerns for risk of bias and serious concerns for imprecision. For the RCT that examined delayed bladder emptying, the certainty of evidence for patient pain was rated as low due to serious concerns for risk of bias, and the certainty of evidence for provider ease of placement and placement success was rated as very low due to serious concerns for risk of bias and serious concerns for imprecision.^33^ For the RCT that examined acupuncture, there were serious concerns with imprecision; the certainty of evidence for patient pain was rated as moderate and the certainty of evidence for need for cervical dilation and placement success was low.^27^ For the RCT examining IUD placement at different menstrual cycle segments, the certainty of evidence for patient pain and provider ease of placement was very low due to serious concerns for risk of bias and serious concerns for imprecision.^26^

## Discussion

### Summary of findings

Our systematic review identified 11 RCTs evaluating various non-pharmacologic techniques to ease IUD placement. The Valsalva manoeuvre, which involves inhaling a deep breath and holding it throughout IUD placement, without use of a tenaculum, was associated with less pain during IUD placement compared with no Valsalva manoeuvre and use of a tenaculum. However, it is unclear whether use of the Valsalva manoeuvre without tenaculum placement is effective at reducing pain compared with no intervention. This study reported similar placement success rates in the intervention and comparison groups and did not assess other outcomes. Additionally, acupuncture was associated with reduced pain 3 min after IUD placement compared with routine care without acupuncture.^27^ This study found similar proportions of need for cervical dilation, placement success and side effects between the acupuncture and control groups. These findings suggest a beneficial impact of the Valsalva manoeuvre without tenaculum placement and acupuncture on reducing patient pain during IUD placement but are based on single studies with relatively small sample sizes and, therefore, should be interpreted with caution. No other pharmacologic technique was found to reduce patient pain.

None of the other interventions were associated with any of the outcomes assessed for this review. Delayed bladder emptying, verbal analgesia and placement during different times of the menstrual cycle did not have a substantial impact on provider ease of IUD placement.^25 26 28 33^ Very few IUD placement failures were reported across these interventions, with no marked differences between study groups. A virtual reality intervention was not associated with increased patient satisfaction. Verbal analgesia, inhaled lavender and placement at different times in the menstrual cycle were not associated with differences in side effects or adverse events compared with controls.

### Strengths and limitations

This review used a comprehensive search strategy to capture a broad range of non-pharmacologic interventions for managing patient pain and facilitating IUD placement. However, the primary limitation of this review is the paucity of high-quality research in this area, with most of the included studies having an overall high risk of bias, being single-centre studies with limited sample sizes and statistical power. Additionally, the heterogeneity in the interventions, comparators and outcomes examined across the included studies precluded conducting a quantitative meta-analysis to synthesise findings. Lack of statistical pooling and integration of the evidence limits our ability to draw robust conclusions from the available evidence. Nonetheless, the review provides a helpful summary of the current state of research on non-pharmacologic techniques to ease interval IUD placement and highlights the need for more rigorous, larger-scale studies to comprehensively evaluate the effectiveness of various non-pharmacologic approaches for reducing patient pain and improving the overall IUD placement experience for women.

### Conclusions and implications for practice

While the current evidence suggests that some non-pharmacologic techniques, specifically the Valsalva manoeuvre without tenaculum placement and acupuncture, may reduce patient pain with IUD placement, the overall quality and quantity of evidence supporting these techniques is limited (certainty of evidence ranging from moderate to low). Further high-quality research is needed to establish more conclusively the effectiveness of these non-pharmacologic techniques for reducing patient pain. Limited evidence suggests that specific non-pharmacologic techniques did not appear to substantially improve provider ease of placement or placement success or reduce the need for cervical dilation (certainty of evidence ranging from low to very low). Nevertheless, many of the non-pharmacologic techniques examined may be easy to implement, are low cost or require few resources, and may provide supportive care to patients, with few side effects and no adverse events reported across the studies. While awaiting more robust evidence, clinicians might consider using a combination of pharmacologic and non-pharmacologic strategies tailored to the individual patient’s preferences and needs to reduce barriers to IUD access and optimise the IUD placement experience.

## Supplementary material

10.1136/bmjsrh-2025-202840 online supplemental file 1

## Data Availability

Data are available upon reasonable request.
