# Supplementary material for: Non-pharmacologic techniques for interval intrauterine device placement: a systematic review
Source: BMJ Sex Reprod Health. 2025 Nov 3;51(Suppl 1):e202840. doi: 10.1136/bmjsrh-2025-202840 (PMC12703282; doi:10.1136/bmjsrh-2025-202840)
Supplement: online supplemental file 1 [file bmjsrh-51-Suppl_1-s001.pdf]

Appendix A.

Search Strategy:

Database	Strategy
**Medline (OVID) 1946-**	1. exp Intrauterine Devices/<br>2. (((Intrauterine OR intra-uterine) ADJ5 (contracept* OR device* OR system*)) OR IUD* OR IUCD* OR IUS OR mirena OR skyla OR paragard OR Copper T380 OR CuT380 OR Copper T380a OR CuT380a OR kyleena OR liletta OR copper 7 OR copper T200B).mp.<br>3. 1 OR 2<br>4. (Insert* OR implant* OR placement OR initiation).mp.<br>5. 3 AND 4<br>6. Randomized controlled trial.pt<br>7. (trial* OR rct* OR random* OR double blind* OR single blind* OR controlled OR control group*).mp<br>8. 6 OR 7<br>9. 5 AND 8
**Embase (OVID) 1974-**	1. exp Intrauterine contraceptive device/<br>2. (((Intrauterine OR intra-uterine) ADJ5 (contracept* OR device* OR system*)) OR IUD* OR IUCD* OR IUS OR mirena OR skyla OR paragard OR Copper T380 OR CuT380 OR Copper T380a OR CuT380a OR kyleena OR liletta OR copper 7 OR copper T200B).mp.<br>3. 1 OR 2<br>4. (Insert* OR implant* OR placement OR initiation).mp.<br>5. 3 AND 4<br>6. (trial* OR rct* OR random* OR double blind* OR single blind* OR controlled OR control group*).mp<br>7. 5 AND 6<br>8. Limit 7 to conference abstract status<br>9. 7 NOT 8<br>10. Limit 9 to remove Medline records
**Cochrane Library – Central**	#1 [mh ^"Intrauterine Devices"]<br>#2 (((Intrauterine OR intra-uterine) NEAR/5 (contracept* OR device* OR system*)) OR IUD* OR IUCD* OR IUS OR mirena OR skyla OR paragard OR “Copper T380” OR CuT380 OR “Copper T380a” OR CuT380a OR kyleena OR liletta OR “copper 7” OR “copper T200B”):ti,ab,kw<br>#3 #1 OR #2<br>#4 (Insert* OR implant* OR placement OR initiation):ti,ab,kw<br>#5 #3 AND #4<br><br>Limit to TRIALS

**WHO Global Index Medicus**	“Intrauterine device” OR “intrauterine system” OR “intrauterine contraceptive” OR “intra-uterine device” OR “intra-uterine system” OR “intra-uterine contraceptive” OR IUD* OR IUCD* OR IUS OR mirena OR skyla OR paragard OR “Copper T380” OR CuT380 OR “Copper T380a” OR CuT380a OR kyleena OR liletta OR “copper 7” OR “copper T200B”<br>AND<br>Insert* OR implant* OR placement OR initiation
**Scopus**	TITLE-ABS-KEY(“Intrauterine device” OR “intrauterine system” OR “intrauterine contraceptive” OR “intra-uterine device” OR “intra-uterine system” OR “intra-uterine contraceptive” OR IUD* OR IUCD* OR IUS OR mirena OR skyla OR paragard OR “Copper T380” OR CuT380 OR “Copper T380a” OR CuT380a OR kyleena OR liletta OR “copper 7” OR “copper T200B”) AND TITLE-ABS-KEY(Insert* OR implant* OR placement OR initiation) AND TITLE-ABS-KEY(trial* OR rct* OR random* OR "double blind*" OR "single blind*" OR controlled OR "control group*") AND NOT INDEX(medline)
**Clinicaltrial.gov**	“Intrauterine device” OR “intrauterine system” OR “intrauterine contraceptive” OR “intra-uterine device” OR “intra-uterine system” OR “intra-uterine contraceptive” OR IUD* OR IUCD* OR IUS OR mirena OR skyla OR paragard OR “Copper T380” OR CuT380 OR “Copper T380a” OR CuT380a OR kyleena OR liletta OR “copper 7” OR “copper T200B” | Insert* OR implant* OR placement OR initiation | Completed Studies
**WHO ICTRP**	“Intrauterine device” OR “intrauterine system” OR “intrauterine contraceptive” OR “intra-uterine device” OR “intra-uterine system” OR “intra-uterine contraceptive” OR IUD* OR IUCD* OR IUS OR mirena OR skyla OR paragard OR “Copper T380” OR CuT380 OR “Copper T380a” OR CuT380a OR kyleena OR liletta OR “copper 7” OR “copper T200B”<br>AND<br>Insert* OR implant* OR placement OR initiation<br><br>With results
